# A Rare Report of Clinical Lycanthropy in Obsessive-Compulsive and Related Disorders

**DOI:** 10.7759/cureus.13346

**Published:** 2021-02-15

**Authors:** Varchasvi Mudgal, Mohd. R Alam, Vijay Niranjan, Priyash Jain, Virendra S Pal

**Affiliations:** 1 Psychiatry, Mahatma Gandhi Memorial Medical College, Indore, IND

**Keywords:** lycanthropy, clinical case report, obsessive compulsive disorders, obsessive-compulsive symptoms

## Abstract

Lycanthropy is a rare variant of a delusional misidentification syndrome specifically reverse inter-metamorphosis where patients believe that they are experiencing transformation or have transformed into an animal. A case report of this phenomenon is discussed. We report the lycanthropy phenomenon of a 25-year-old male who believed himself to be transformed into a buffalo after bestiality along with developing obsessive-compulsive features. A case report along with a literature review forms the basis of discussion. Clinical lycanthropy has been reported with various neuropsychiatric conditions including primary psychotic and affective conditions, drug intoxication and withdrawal, cerebrovascular disease, traumatic brain injury, dementia, delirium, and seizures, but its association in the context of obsessive-compulsive and related disorders (OCRDs) is a very rare finding. A differential of OCRD should be given due importance and managed accordingly when dealing with lycanthropy, which may help in early identification and management.

## Introduction

Lycanthropy is an uncommon form of delusion or unusual belief that one has transformed into a non-human animal or displays behaviour or feelings indicative of such a belief. It is a rare variant of delusional misidentification syndrome (DMS) specifically reverse inter-metamorphosis where patients believe that they are experiencing transformation or have transformed into an animal. DMS is seen in association with several neuropsychiatric conditions, including primary psychotic and affective conditions, drug intoxication and withdrawal, cerebrovascular disease, traumatic brain injury, dementia, delirium, and seizures [1]. A “two-factor theory” has been proposed for lycanthropy where the primary trigger for delusion formation likely involves a mismatch in the individual’s neural representation of his “Self.” The second factor is well-thought-out to be impairment in the belief-evaluation system that averts the delusional explanation from being rejected [2]. Lycanthropy does not form a distinct syndrome but is a symptom of different psychiatric illnesses. It is largely influenced by the socio-cultural environment of the patient so that the animal species often represents the patient’s delusional depiction of evil. A case of such a delusional misidentification involving the self in a 25-year-old milkman suffering from obsessive-compulsive and related disorder (OCRD) and had done the act of bestiality with one of his own buffaloes is presented here.

## Case presentation

**Figure 1:**
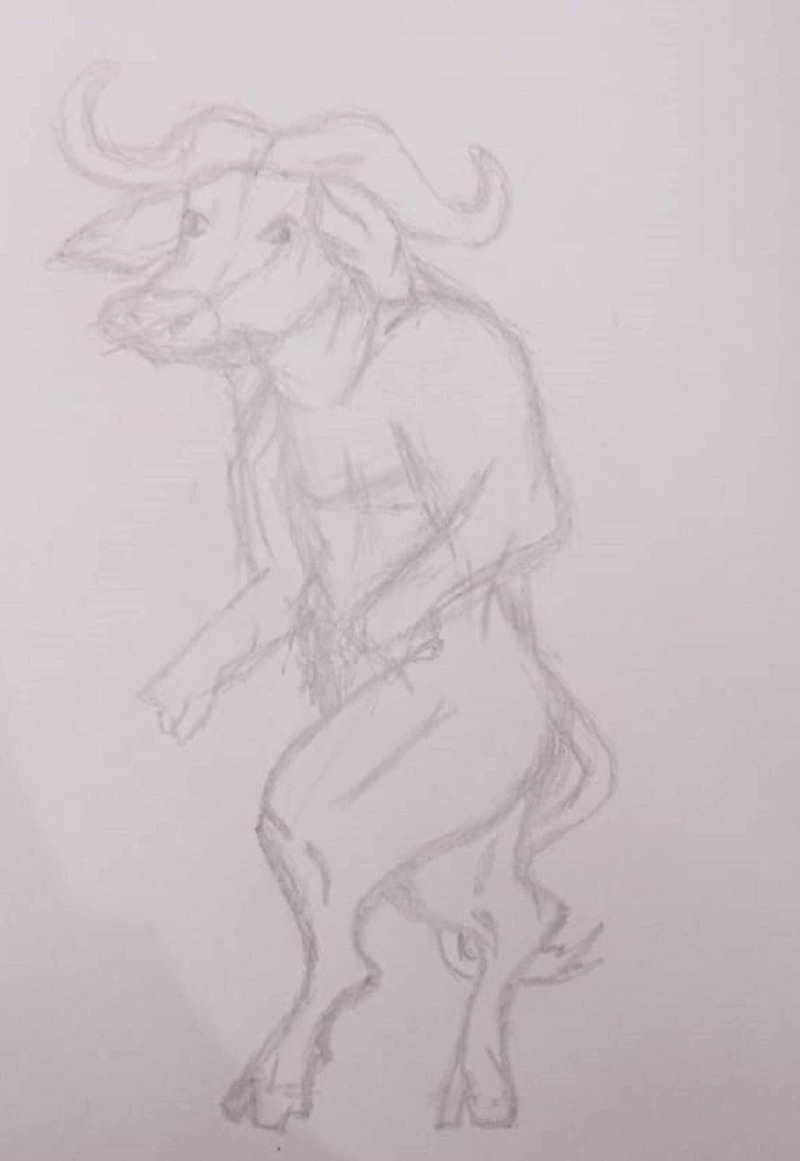
Representation of body image as drawn by the patient. Consent was obtained for publishing the drawing from the patient.

The patient was a 25-year-old milkman from a low socio-economic class and residing in an urban area. The patient was brought to the Department of Psychiatry by family members with complaints of excessive hand washing, irritable behaviour and decreased sleep along with recurrent cleaning of genital areas, and acting like a buffalo for four months. During an interview with the patient, he reported engaging in sexual activity with his own buffalo a few times six months ago and since then he believes that a few cells of the buffalo have entered into his body and he would turn in a buffalo. To avoid this, he recurrently washes his hands and genitals. Gradually his belief strengthened, and he was convinced that he had become a buffalo due to his bestiality. Along with this, his obsession with contamination and compulsive hand washing continued. About two months after the act of bestiality, he started believing his body had transformed into a buffalo beginning from his lower half towards torso and face. When he looked at himself or in the mirror, he visualized his body parts like a buffalo and kept on screaming about his repugnant state and was constantly preoccupied about his looks (Figure 1). However, his family members did not see any visible change in his body, despite that the patient was unconvinced when reassured and repeatedly tried to scrub his body and looked in the mirror to see if he reverted back to himself. As per his family members for the last few weeks, he had started to act like a buffalo by nodding his head, walking on all four limbs, and asking for hay and grass to eat. This buffalo-like behaviour would recur throughout the day. He would occasionally curse and beat the buffalo on which he had performed bestiality as he believed it to be the reason for his transformation. The family members took the patient for faith healing two weeks before bringing him to the current psychiatric facility where some rituals were performed; however, his behaviour did not improve. Afterwards, the family members brought the patient to the hospital.

A diagnosis of obsessive-compulsive disorder (OCD) and body dysmorphic disorder with delusional beliefs was formulated as per Diagnostic and Statistical Manual of Mental Disorders, 5th Edition (DSM V). As per Yale-Brown Obsessive-Compulsive Scale (YBOCS), the obsession score was 17 and compulsion score was 15 (YBOCS total score of 32) [3]. Computed tomography scan was done to rule out organic causes. Routine investigations were performed in the National Accreditation Board for Testing and Calibration Laboratories accredited central lab of our tertiary care hospital, which were unremarkable. Pharmacotherapy included fluoxetine 20 mg and titrated up to 60 mg in four weeks along with augmentation with risperidone 2 mg titrated up to 4 mg. After six months of pharmacotherapy along with the help of family support and drug compliance, his delusional belief of body dysmorphia reduced, and his hand washing was minimal.

## Discussion

Lycanthropy is a rare psychiatric phenomenon seen in the context of various neuropsychiatric conditions and not a disorder itself. Its differentials include schizophrenia, delusional disorder, bipolar disorder, psychotic depression, epilepsy, organic causes; however, its presence in the context of OCRDs is rare. It can be viewed as a delusional misidentification of own body parts [4]. The presence of repeated behaviour along with dysmorphia, which is an OCD spectrum disorder along with the response to high-dose selective serotonin reuptake inhibitor (SSRI) supports our diagnosis. Bestiality is a type of sexual offence in which animal is used as a medium for satisfying sexual desire without developing any kind of emotional bonding [5]. In concordance with reports in the literature of similarly reported cases, the patient had onset of lycanthropy after sexual interaction with an animal (bestiality), which led to obsessive thoughts about turning into an animal that finally gave way to delusional belief of animal transformation and related behaviour. The patient’s walking like the four-legged animals, asking for cattle fodder, and typical head movements mimicking buffalo were consistent with the earlier work [6]. However, he did not have any history of animal bites nor a connection between the full moon and the emergence of the symptoms of the disease [7]. The patient presumed his body to be repugnant to self and others along with constant preoccupation about his body representing dysmorphophobic characteristics. The earlier reports do not refer to any link between the sexual paraphilia and lycanthropy. Interestingly, bestiality was the precipitating cause for our case and was vital in the onset of OCD and related symptoms. As per existing literature delusion may or may not be under the patient’s control. However, in this case, the patient could not exert any control over his transformation. It has been argued in some studies that the lycanthropy syndrome could not affect the prognosis of the previously dominant disease as was the case in the present report. The symptoms of lycanthropy observantly decreased within six months; however, his hand washing and obsessions although reduced but persisted nevertheless similar to what has been reported in the literature [8]. Lycanthropy is usually considered the sine qua non of a small section of neuropsychiatric disorders backed by earlier reports [9,10].

## Conclusions

The lycanthropy phenomenon has been reported in various major psychiatric disorders such as schizophrenia, delusional disorders, affective disorders, and other organic states. However, cases of secondary clinical lycanthropy in OCRD are rare. We urge the clinicians to also consider clinical lycanthropy a symptom secondary to OCRDs, which should be given due importance and which may help in early identification and management.
